# Proteomic Alterations in Hypomineralised Enamel: A Systematic Review

**DOI:** 10.3390/dj14100681

**Published:** 2026-10-19

**Authors:** Gabriela Balixa, Bernhard Ganss, Luísa Bandeira Lopes

**Affiliations:** 1 Egas Moniz Center for Interdisciplinary Research (CiiEM), Egas Moniz School of Heath & Science, Campus Universitário, Quinta da Granja, 2829-511 Caparica, Almada, Portugal; gbalixa@egasmoniz.edu.pt; 2 Faculty of Dentistry, University of Toronto, Toronto, ON M5G 1G6, Canada

**Keywords:** molar incisor hypomineralisation, hypomineralised second primary molars, proteomics, enamel, saliva, biomarkers, systematic review

## Abstract

**Background:** Developmental enamel hypomineralisation is characterised by qualitative defects in enamel mineralisation that affect both primary and permanent dentitions. Proteomic studies have provided novel insights into the molecular mechanisms underlying these conditions; however, an updated critical appraisal integrating the available evidence across both Molar–Incisor Hypomineralisation (MIH) and Hypomineralised Second Primary Molars (HSPM) is needed. **Methods:** A systematic review was performed in accordance with the Preferred Reporting Items for Systematic Reviews and Meta-Analyses PRISMA 2020 guidelines. PubMed/MEDLINE, Embase, Scopus, and Web of Science were searched from inception until 30 July 2026. Observational and laboratory studies examining proteomic changes in enamel, saliva, or other oral biological samples from individuals diagnosed with MIH or HSPM were included. Risk of bias was assessed using the revised Joanna Briggs Institute (JBI) tool and the Quality Assessment Tool for In Vitro Studies (QUIN). **Results:** Ten reports satisfied the inclusion criteria, representing nine independent primary datasets. Consistent findings included increased retention of serum-derived proteins, particularly albumin, alongside enrichment of protease inhibitors and inflammatory proteins within hypomineralised enamel. Additionally, alterations in proteins involved in enamel matrix processing and maturation, including matrix metalloproteinase-20 (MMP20) and kallikrein-related peptidase 4 (KLK4), were identified. Salivary analyses revealed quantitative differences in proteins involved in immune, inflammatory and mineralisation-related pathways. Both shared and distinct proteomic features were observed across hypomineralised enamel in the primary and permanent dentitions. **Conclusions:** Current evidence indicates recurrent proteomic alterations in developmental enamel hypomineralisation, particularly impaired enamel maturation, abnormal retention of serum-derived proteins, and alterations in inflammatory pathways. Further standardised proteomic studies are needed to validate these findings and assess their potential as biomarkers.

## 1. Introduction

Developmental enamel hypomineralisation comprises qualitative defects of enamel mineralisation resulting from disturbances during amelogenesis and affecting both the primary and permanent dentitions. Molar–incisor hypomineralisation (MIH) is characterised by demarcated opacities involving one or more first permanent molars, frequently associated with permanent incisors, whereas hypomineralised second primary molars (HSPM) affect the primary dentition and are considered an important predictor of MIH [1,2,3].

Terminology varies across the included literature. Most studies use the terms MIH or HSPM, whereas Williams et al. [1] use the term molar hypomineralisation (MH). The National Library of Medicine defines MH as a developmental disturbance of amelogenesis involving molars of any type, with possible involvement of other teeth, characterised by demarcated enamel opacities of unknown systemic causation [2]. Accordingly, the terminology used by the original authors is retained when describing individual studies.

Hypomineralisation primarily reflects disturbances during the maturation stage of amelogenesis, resulting in enamel of reduced mineral quality but relatively preserved thickness, whereas disturbances during the secretory stage may result in quantitative defects such as enamel hypoplasia [4].

Clinical manifestations range from white–cream or yellow–brown opacities to post-eruptive enamel breakdown, dentine hypersensitivity, rapid caries progression and restoration failure, with a significant impact on oral health-related quality of life [5].

MIH affects approximately one in seven children worldwide, with a pooled global prevalence of 13.5% reported in a recent meta-analysis [6], consistent with previous global estimates [7,8]. Despite extensive epidemiological research, its aetiology remains incompletely understood. Prenatal, perinatal and postnatal exposures, including systemic illnesses, medication use, environmental factors and genetic susceptibility, have been proposed, but no single factor consistently explains its development, supporting a multifactorial origin [6,9]. Importantly, substantial gaps remain in understanding the biological mechanisms underlying enamel hypomineralisation [6].

Proteomic research has provided new insights into these mechanisms. Early investigations demonstrated substantially increased protein content in hypomineralised enamel, including retention of serum-derived proteins such as albumin, α1-antitrypsin and antithrombin III [10,11]. Subsequent high-resolution proteomic studies identified alterations in proteins associated with enamel maturation, inflammatory and immune responses, extracellular matrix organisation, mineralisation and cellular stress [12,13,14,15]. In one study, MMP20 and KLK4 were comparatively more abundant in sound enamel than in MIH enamel [13]. Salivary analyses also suggested potential non-invasive biomarker signatures [12,14]. These findings have also provided molecular support for pathogenetic hypotheses such as the Mineralisation Poisoning Model, in which serum albumin is proposed to interfere with normal crystal growth during enamel maturation [16,17].

Despite the increasing number of proteomic investigations, the available evidence remains fragmented due to heterogeneity in biological samples, analytical platforms and reported outcomes. A recent scoping review mapped the available evidence on enamel and salivary proteomics in MIH, including six studies identified up to February 2025 [18]. However, the review was restricted to MIH and did not formally appraise the methodological quality or risk of bias of the included studies. Since then, additional proteomic evidence has become available, including studies of HSPM, additional salivary proteomic investigations and direct comparative analysis of HSPM and MIH proteomic profiles [19,20,21,22]. Consequently, the available literature lacks a systematic and methodologically appraised synthesis integrating proteomic evidence from both MIH and HSPM across primary and permanent dentitions. Such an analysis is needed to determine whether recurrent proteomic patterns are consistent across studies and biological samples, to critically evaluate the strength and methodological limitations of the available evidence, and to assess whether recent findings provide additional insight into potential pathogenic mechanisms and biomarker applications. Therefore, this systematic review aimed to identify and critically synthesise proteomic alterations associated with developmental enamel hypomineralisation, evaluate the methodological quality of the available evidence, examine their potential as biomarkers and their contribution to the understanding of underlying pathogenic mechanisms, and explore similarities and differences across affected primary and permanent dentitions.

## 2. Materials and Methods

### 2.1. Protocol and Registration

The protocol for this systematic review was developed by all authors and registered with the International Prospective Register of Systematic Reviews (PROSPERO; registration number: CRD420261471486). The terminology used for molar hypomineralisation was also considered in accordance with the Medical Subject Headings (MeSH) definition provided by the National Library of Medicine [23].

This systematic review was conducted and reported in accordance with the Preferred Reporting Items for Systematic Reviews and Meta-Analyses (PRISMA 2020) statemement [24,25]. The completed PRISMA 2020 checklist is provided as Supplementary Material Table S5.

### 2.2. Focused Question and Eligibility Criteria

We developed a protocol to answer the following Population, Exposure, Comparator, Outcome (PECO) question: “What proteomic alterations have been identified in enamel and salivary samples from individuals with MIH or HSPM compared with healthy controls or across different severity grades, and how do these findings contribute to the understanding of their pathogenesis?” The eligibility criteria were established according to the PECO (Population, Exposure, Comparator, Outcome) framework.

Population: Individuals diagnosed with MIH and/or HSPM, including studies using the term MH for clinically corresponding hypomineralisation phenotypes.

Exposure: Proteomic analysis of enamel, saliva or other oral biological samples from individuals with MIH or HSPM.

Comparator: Healthy enamel or saliva, and/or comparisons between different severity grades of MIH or HSPM.

Outcome: Differentially expressed proteins, proteomic biomarkers, biological pathways and implications for the pathogenesis of MIH and HSPM.

Studies were considered eligible if they met all of the following criteria: (1) original peer-reviewed analytical observational or laboratory studies; (2) conducted using human participants or biological samples obtained from individuals with MIH or HSPM, including studies using MH terminology for clinically corresponding hypomineralisation phenotypes; (3) investigated proteomic alterations associated with these conditions; (4) analysed enamel, saliva or other relevant oral biological samples; (5) included healthy controls and/or comparisons according to defect severity; and (6) employed recognised proteomic or protein analytical methodologies, such as liquid chromatography–tandem mass spectrometry (LC–MS/MS), matrix-assisted laser desorption/ionisation time-of-flight mass spectrometry (MALDI-TOF MS), sodium dodecyl sulphate–polyacrylamide gel electrophoresis (SDS-PAGE), two-dimensional gel electrophoresis, Western blotting or equivalent techniques.

The exclusion criteria comprised: (1) reviews; (2) systematic reviews; (3) umbrella reviews; (4) scoping reviews; (5) conference abstracts; (6) editorials; (7) letters; (8) case reports; (9) animal studies; (10) in vitro or laboratory studies that did not analyse biological samples obtained from individuals with eligible hypomineralisation phenotypes; (11) studies investigating restorative materials without proteomic or protein analysis; (12) studies evaluating other developmental enamel defects without reporting separate data for the eligible hypomineralisation phenotypes; and (13) studies that did not report original proteomic data. No restrictions were applied regarding language or year of publication.

### 2.3. Search Strategy and Study Selection

The final database searches were conducted on 30 July 2026 by two independent reviewers (GB and LBL) in the following databases: PubMed via MEDLINE, Scopus, Web of Science Core Collection, and Embase. The reference lists of all included studies and relevant review articles were manually screened to identify additional eligible reports. The search strategy combined controlled vocabulary (MeSH and Emtree terms) with free-text terms related to MIH and proteomics. Database-specific search syntax was adapted to the indexing structure and search functionalities of each database, and the complete search strategies are provided in Supplementary Table S1.

The search results were uploaded into Rayyan (web-based platform; accessed 30 July 2026), a web-based systematic review management platform [26], and a second check for duplicates was performed. DedupEndNote (Version 1.0.0) was used for automated deduplication [27]. Titles and abstracts were independently screened by two reviewers (GB and LBL), followed by independent full-text assessment of potentially eligible studies. Disagreements were resolved through discussion and, when necessary, consultation with a third reviewer (BG).

### 2.4. Data Extraction Process and Data Items

Data extraction was performed independently and in duplicate by two reviewers (GB and LBL). Disagreements were resolved through discussion and, when necessary, consultation with a third reviewer (BG). The following information was extracted: first author, publication year, country, study design, sample characteristics, condition and terminology used in the original study (MIH, HSPM or MH, as applicable), sample type (enamel, saliva or dentine–enamel junction), sample size, participant age, diagnostic criteria, severity classification, control group characteristics, analytical methodology and platform, proteomic findings, differentially abundant proteins, biological pathways, principal findings and funding.

### 2.5. Risk of Bias Assessment

The risk of bias of the included studies was independently assessed by two reviewers (GB, LBL) according to study design. Clinical observational studies were evaluated using the revised JBI critical appraisal tool for the assessment of risk of bias for analytical cross-sectional studies [28], whereas laboratory studies performed on biological samples obtained from affected individuals were assessed using the Quality Assessment Tool for In Vitro Studies (QUIN) [29].

For the revised JBI tool, risk of bias was assessed across the relevant domains according to the guidance provided by JBI, with judgements made at the item level and an overall qualitative assessment based on the pattern and relevance of identified concerns. No numerical summary score was calculated. For the QUIN tool, each applicable criterion was scored according to the original scoring system, and the overall score was calculated as a percentage and classified as low (>70%), medium (50–70%), or high (<50%) risk of bias.

Disagreements between reviewers were resolved through discussion and, when necessary, by consultation with a third reviewer (BG). The complete risk-of-bias assessment is provided in Supplementary Table S3.

### 2.6. Data Synthesis

Given the heterogeneity in biological samples, proteomic methodologies and reported outcomes, quantitative meta-analysis was not considered appropriate. Findings were therefore synthesised qualitatively, with studies grouped according to biological sample, condition and reported proteomic patterns.

Given the inclusion of both discovery-based proteomic approaches and targeted protein-detection methods, findings were synthesised according to the analytical scope of each study. Targeted studies were considered as evidence for the presence or relative detection of prespecified proteins and were not treated as equivalent to discovery-based proteomic investigations in terms of proteome coverage or capacity to identify differentially abundant proteins.

The synthesis focused on recurrent alterations involving serum-derived proteins, enamel matrix and maturation-related proteins, protease–antiprotease balance, inflammatory and immune-related proteins, extracellular matrix and cellular processes, mineralisation-related pathways, severity-associated proteomic differences and salivary proteomic profiles. Similarities and differences across affected primary and permanent dentitions were also explored.

Following the qualitative synthesis, the findings were interpreted in relation to current mechanistic hypotheses of enamel hypomineralisation to explore potentially convergent biological mechanisms, assess the consistency of the available proteomic evidence and highlight current knowledge gaps.

## 3. Results

### 3.1. Study Selection

**Figure 1:**
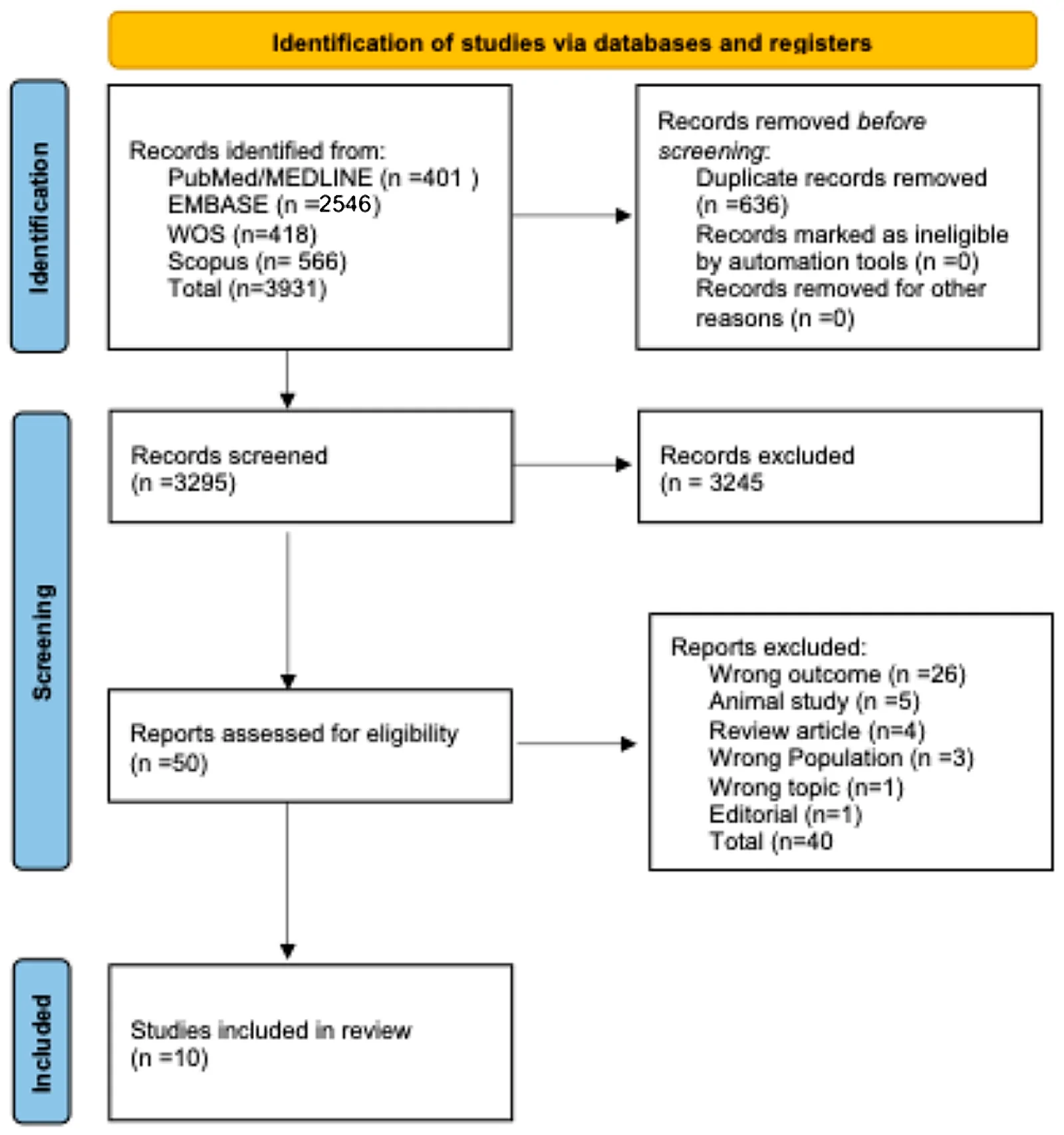
PRISMA Flowchart.

The database search identified 3931 records. After removal of 636 duplicates, 3295 records remained for title and abstract screening, of which 3245 were excluded. Fifty full-text articles were assessed for eligibility. Following full-text evaluation, 40 studies were excluded. The complete list of exclusion reasons is provided in the Supplementary Table S2. Consequently, ten reports fulfilled the eligibility criteria and were included in the qualitative synthesis, representing nine independent primary datasets (Figure 1).

### 3.2. Characteristics of the Included Studies

The ten included reports were published between 2010 and 2026 and were conducted across seven countries: New Zealand, Australia, India, Sweden, Austria, Greece, and Brazil [10,12,13,14,15,16,19,20,21,22]. Seven reports investigated enamel proteomics, whereas three evaluated salivary proteomes. Of the seven enamel reports, Singh et al. [22] was a secondary comparative proteomic analysis of previously generated datasets from Singh et al. [20] and Mukhtar et al. [13], rather than an independent sample. Sample sizes varied considerably across the primary studies, ranging from small laboratory series to clinical cohorts of up to 50 participants, with reported participant ages ranging from 4 to 18 years. General characteristics of the included reports, including study design, sample characteristics, diagnostic criteria and severity classification, are presented in Table 1.

### 3.3. Risk of Bias

The three observational studies assessed using the revised JBI risk-of-bias tool for analytical cross-sectional studies showed few methodological concerns overall [12,14,21]. The main concerns related to the identification and management of potential confounding factors and, where applicable, the reporting of strategies used to address them. The seven laboratory-based reports assessed using QUIN were all classified as having a medium risk of bias, with scores ranging from 54.2% to 66.7%: Farah et al. [10], 54.2%; Williams et al. [16], 54.2%; Mukhtar et al. [13], 62.5%; Rexhaj et al. [15], 58.3%; Sharon et al. [19], 66.7%; Singh et al. [20], 66.7%; and Singh et al. [22], 62.5%. The main limitations related to sample-size calculation, randomisation, blinding and operator-related reporting. Detailed assessments are presented in Supplementary Table S3.

### 3.4. Proteomic Findings

#### 3.4.1. Enamel Proteomics

Seven reports investigated the proteomic composition of hypomineralised enamel using complementary analytical approaches [10,13,15,16,19,20,22]. Across studies, hypomineralised enamel consistently showed increased protein content and retention of serum-derived proteins compared with sound enamel, with albumin representing the most recurrent finding. Alterations in protease inhibitors, including α1-antitrypsin and antithrombin III, were also reported, together with alterations in proteins involved in enamel matrix processing and maturation.

Proteins involved in physiological enamel formation and maturation also differed between hypomineralised and sound enamel. In Mukhtar et al. [13], MMP20 and KLK4 were more abundant in sound enamel, while other studies reported alterations in enamel matrix-related proteins, including ameloblastin and amelogenin [10,20]. Additional alterations involved immune and inflammatory responses, complement activation, extracellular matrix organisation and mineralisation-related processes. Severity-associated differences in proteomic profiles were also identified in some enamel samples. Overall, recurrent alterations involved serum-derived protein retention, protease–antiprotease balance and enamel matrix processing across hypomineralised enamel from affected primary and permanent teeth. Singh et al. [22] performed a secondary comparative analysis of previously generated HSPM and MIH proteomic datasets. A total of 435 proteins were identified across the two datasets, including 120 proteins shared between HSPM and MIH, of which 90 were retained for quantitative comparison. Forty-six proteins differed significantly in abundance (FDR < 0.05), with 38 enriched in HSPM and eight in MIH. HSPM showed relative enrichment of immune/plasma and protease–antiprotease-related proteins, with pathways involving acute-phase and inflammatory responses, complement/coagulation and platelet/neutrophil degranulation. In contrast, MIH showed greater enrichment of epithelial and cornification-related proteins and pathways involving keratinocyte differentiation, cytoskeletal organisation and the cornified envelope. Overall, the direct comparison indicated a largely shared enamel proteome but distinct quantitative profiles between HSPM and MIH.

#### 3.4.2. Salivary Proteomics

Three studies investigated salivary proteomic alterations in affected children [12,14,21]. Despite methodological differences, distinct proteomic profiles were consistently reported, involving proteins related to innate immunity, inflammatory responses, complement activation, extracellular matrix organisation and calcium-related processes. Severity-associated differences were also identified in one study [21]. Detailed proteomic methodologies and findings of the individual studies are presented in Table 2.

#### 3.4.3. Integrated Proteomic Patterns

Given the biological and methodological differences between enamel and saliva, findings from these sample types were interpreted separately and were not considered to represent a single proteomic signature. Enamel findings primarily reflected proteins retained within or associated with the hypomineralised tissue, whereas salivary findings reflected a broader oral biological environment and may be influenced by local and systemic factors.

Across the included reports, recurrent proteomic alterations could be grouped into serum-derived protein retention, protease–antiprotease imbalance, altered enamel matrix processing, immune- and inflammatory-related alterations, complement/coagulation pathways, mineralisation-related processes and extracellular matrix/cellular alterations. Overlapping molecular features were observed across affected primary and permanent dentitions; however, direct comparative evidence also identified distinct quantitative proteomic profiles between HSPM and MIH, particularly involving immune/plasma and protease–antiprotease-related proteins in HSPM and epithelial/cornification-related proteins in MIH [22]. Some studies additionally identified severity-associated proteomic differences. An integrated synthesis of these proteomic patterns and their potential biological relevance is provided in Supplementary Table S4.

Owing to substantial heterogeneity in biological samples, analytical methodologies and outcome reporting, quantitative synthesis was not considered appropriate.

## 4. Discussion

### 4.1. Principal Findings

This systematic review extends the previous scoping synthesis of proteomic alterations in MIH by providing an updated and methodologically appraised evaluation of the evidence across both MIH and HSPM. Whereas the previous scoping review included six studies restricted to MIH, the present review incorporates ten reports, expands the evidence base to affected primary and permanent dentitions, includes three salivary proteomic investigations, and formally evaluates methodological quality and risk of bias using design-specific tools. Importantly, recent evidence also enables direct comparison of HSPM and MIH proteomic profiles, revealing a substantial shared protein repertoire alongside distinct quantitative patterns [22]. Despite the limited evidence base and substantial methodological heterogeneity, recurrent alterations were identified involving serum-derived protein retention, protease–antiprotease balance, enamel matrix processing and maturation, and immune- and inflammatory-related pathways.

The most consistent finding was the retention of serum-derived proteins, particularly albumin, in hypomineralised enamel, accompanied by alterations in protease inhibitors and proteins involved in enamel matrix processing and maturation [10,13,15,16,19,20]. Salivary studies additionally identified dysregulation of immune, inflammatory, extracellular matrix and mineralisation-related pathways [12,14,21].

Collectively, these findings suggest that developmental enamel hypomineralisation involves disturbances in enamel maturation rather than a single molecular abnormality. The recurrence of related proteomic patterns across different populations, biological samples and affected dentitions strengthens their potential biological relevance. However, the limited number of studies, differences in analytical approaches and predominantly observational or laboratory-based evidence preclude causal inference.

### 4.2. Serum-Derived Proteins and Impaired Enamel Maturation

Albumin emerged as the most consistently identified serum-derived protein in hypomineralised enamel across the included studies [10,13,15,16,19,20]. This finding is biologically relevant because mature enamel normally contains minimal residual protein following degradation and removal of the organic matrix during maturation [30,31]. The persistence of serum-derived proteins may therefore reflect disruption of normal enamel maturation.

Several observations further support this hypothesis. Increased protein content and severity-associated differences have been reported in hypomineralised enamel [10,15], while the detection of α-fetoprotein by Williams et al. [16] suggests that at least part of the retained protein may be incorporated during tooth development rather than exclusively after eruption. Experimental evidence suggests that albumin can be incorporated into developing enamel and may inhibit crystallite growth [32]. These observations are compatible with the Mineralisation Poisoning Model, which proposes that serum albumin entering immature enamel may interfere with normal mineral crystal growth during maturation [17]. Albumin is a serum-derived protein that is normally largely excluded from the developing enamel compartment by the cellular layers separating the enamel space from the adjacent vascularised tissues. During enamel maturation, alterations in the enamel organ may affect its barrier function, potentially facilitating the entry of serum-derived proteins into the developing enamel. The presence of albumin within intact hypomineralised enamel may therefore indicate altered integrity or permeability of this physiological barrier during enamel development, allowing serum proteins to access the mineralising enamel matrix. Once incorporated, albumin has been proposed to interfere with hydroxyapatite crystal growth, potentially contributing to defective mineralisation [16,32,33].

The recurrent detection of albumin in hypomineralised enamel represents an association consistently identified across several proteomic studies. Experimental observations showing that albumin can bind to hydroxyapatite and interfere with crystal growth provide biological plausibility for the Mineralisation Poisoning Model. However, these findings do not establish albumin as a primary initiating factor in MIH or HSPM, and the current evidence is insufficient to demonstrate a direct causal relationship.

The proteomic evidence also indicates alterations in the mechanisms responsible for organic matrix removal. MMP20 and KLK4, which contribute to the processing and removal of enamel matrix proteins during amelogenesis [34,35], were comparatively more abundant in sound enamel in Mukhtar et al. [13], while Singh et al. [20] reported greater abundance of several matrix- and mineralisation-related proteins in control enamel, while alterations in protease inhibitors, including α1-antitrypsin and antithrombin III, were reported in hypomineralised samples [10,19]. Together, these findings suggest that abnormal protein retention may reflect an imbalance between matrix processing, proteolytic activity and mineral deposition rather than an alteration attributable to a single protein or pathway.

However, the direction and functional significance of individual protein alterations were not uniform across studies [10,13,15,19,20]. The available evidence therefore cannot establish whether serum-protein retention represents a primary pathogenic event or occurs secondary to other disturbances in enamel maturation. Functional studies combining quantitative proteomics with assessment of protease activity and temporally relevant models of amelogenesis, or even calcium phosphate mineralization in vitro, will be required to clarify these relationships.

### 4.3. Inflammatory Pathways and Salivary Proteomics

The three salivary studies identified distinct proteomic profiles involving innate immunity, inflammatory responses, complement activation, extracellular matrix organisation and calcium-related processes [12,14,21]. These findings broaden the molecular perspective beyond the structural abnormalities identified directly in enamel and suggest that affected individuals may exhibit alterations in local or systemic biological pathways.

Interpretation of these findings nevertheless requires caution. Saliva reflects multiple local and systemic processes, and its proteomic composition may be influenced by caries, gingival inflammation, plaque accumulation and post-eruptive changes associated with porous hypomineralised enamel. Consequently, the inflammatory and immune-related signatures identified in affected children cannot currently be assumed to represent mechanisms involved directly in defect development [12,14,21]. The microbiome differences reported by Pappa et al. [14] further suggest a potential interaction between the enamel phenotype, oral microbial environment and host response, although microbiome analysis was beyond the scope of the present review.

Salivary proteomics nevertheless represents a potentially valuable approach for identifying non-invasive molecular markers, particularly in paediatric populations due to the relative ease of sample collection. Severity-associated proteomic differences reported in some studies further support investigation of this possibility [21]. However, no protein or proteomic signature identified to date can be considered a validated diagnostic or prognostic biomarker. Standardised sampling protocols, harmonised analytical methods, larger cohorts and independent validation will therefore be essential before clinical translation can be considered [12,14,21].

### 4.4. Biological Convergence Across Affected Dentitions

An important finding of this review was the presence of both shared and distinct proteomic features across affected primary and permanent dentitions. Previous studies independently identified retention of serum-derived proteins and alterations involving protease activity, matrix processing and mineralisation-related pathways in both HSPM and MIH [10,13,15,16,19,20]. More recently, direct comparison of HSPM and MIH proteomic datasets demonstrated a largely shared protein repertoire but distinct quantitative profiles, with HSPM showing relative enrichment of immune/plasma and protease–antiprotease-related proteins and MIH greater enrichment of epithelial and cornification-related proteins [22]. These findings provide more direct molecular evidence of biological overlap between affected dentitions while also indicating potentially relevant differences in their proteomic profiles.

However, these findings should not be interpreted as demonstrating that defects affecting primary and permanent dentitions represent the same clinical entity or necessarily share an identical aetiology. Differences in the timing of enamel maturation and in prenatal, perinatal and postnatal exposure windows may result in distinct biological responses. Moreover, proteomic evidence involving affected primary teeth remains limited compared with that available for the permanent dentition. The current evidence therefore supports the presence of overlapping molecular features across affected dentitions, while conclusions regarding a common underlying pathogenesis remain premature.

Further investigation of the primary dentition may be particularly informative because the enamel in second primary molars matures earlier than that in first permanent molars and may therefore reflect disturbances occurring during different developmental windows [2,36]. Comparative longitudinal studies with standardised proteomic methodologies across primary and permanent teeth could help determine which molecular alterations are shared and which may be dentition-specific.

### 4.5. Strengths, Limitations and Future Directions

Several limitations should nevertheless be acknowledged. Only ten reports fulfilled the eligibility criteria, and these did not represent ten fully independent datasets, as Singh et al. [22] performed a secondary comparative analysis of previously published HSPM and MIH datasets. Moreover, most primary studies included relatively small sample sizes, and substantial heterogeneity existed in biological specimens, analytical platforms, protein identification approaches, diagnostic criteria and outcome reporting. These differences precluded quantitative synthesis and limit direct comparison between studies.

The included studies also differed substantially in analytical scope. Discovery-based mass-spectrometry approaches enable broad, relatively unbiased characterisation of the proteome, whereas targeted techniques such as Western blotting assess prespecified proteins and provide more restricted proteomic coverage. Consequently, recurrence of individual proteins across studies employing different analytical strategies should be interpreted as convergent evidence of their detection or altered abundance rather than as directly comparable quantitative proteomic findings.

In addition, the available evidence was derived from observational or laboratory-based investigations, preventing causal inference. Risk-of-bias assessment identified some concerns regarding the identification and management of potential confounding factors in the observational studies, while the laboratory-based studies showed limitations particularly related to sample-size calculation, randomisation, blinding, and operator-related reporting. Another limitation relates to the interpretation of proteomic alterations across biological samples. Proteins detected in enamel may more directly reflect processes associated with enamel formation and maturation, whereas salivary proteins can originate from multiple local and systemic sources and may be affected by secondary oral conditions. Accordingly, convergence between enamel and salivary findings should be interpreted as hypothesis-generating rather than evidence of a single shared biological mechanism [12,14,21].

Future research should prioritise adequately powered multicentre studies using standardised diagnostic criteria, sample collection procedures and proteomic workflows [37]. Replication of candidate proteins in independent populations, quantitative validation and functional investigation of the pathways identified will be particularly important. Integration of proteomic findings with genomic, transcriptomic and metabolomic approaches may ultimately provide a more comprehensive understanding of the biological mechanisms underlying developmental enamel hypomineralisation and clarify whether molecular signatures have diagnostic or prognostic value.

## 5. Conclusions

Current proteomic evidence identifies recurrent alterations in developmental enamel hypomineralisation, particularly involving serum-derived protein retention and enamel matrix processing and maturation. Both shared and distinct proteomic features have been reported across affected primary and permanent dentitions, although the limited number of independent datasets and substantial methodological heterogeneity preclude causal conclusions. These findings improve understanding of the biological basis of MIH and HSPM; however, for practising dentists, the current evidence does not yet support the use of proteomic biomarkers for diagnosis, risk assessment or clinical management. Further standardised, adequately powered and independently validated proteomic studies are required.

## Figures and Tables

**Table 1:** General characteristics of the included studies.

Author (Year)	Country	Study Design	Dentition, Teeth and Sample Characteristics	Condition (MIH/HSPM/MH)	Sample type	Sample Size	Participant Age	Diagnostic Criteria	Severity Classification
Enamel proteomics									
Farah et al. [10]	New Zealand	In vitro comparative	Permanent dentition; first permanent molars affected by MIH and sound enamel	MIH	Enamel	10 MIH; 6 controls	7.5–10.5	Clinical diagnosis; Chawla classification	White, yellow, brown
Williams et al. [16]	Australia	In vitro laboratory study	Permanent dentition; first permanent molars affected by MIH	MH	Enamel	15 opacities from 6 molars (6 cases); 8 opacities from 5 cases analysed for ALB/AFP	NR	Standard criteria for demarcated opacities	NR
Mukhtar et al. [13]	India	In vitro comparative study	Permanent dentition; first permanent molars affected by MIH and sound enamel	MIH	Enamel	10 MIH; 10 controls	NR	Weerheijm criteria (2003)	White, yellow, brown
Rexhaj et al. [15]	Sweden	In vitro comparative study	Permanent dentition; first permanent molars affected by MIH and sound enamel	MIH	Enamel	Controls (n = 3); MIH I (white/creamy opacities, n = 4); MIH II (yellow/brown opacities, n = 4)	6–18	EAPD criteria	Clinical grades
Sharon et al. [19]	India	In vitro comparative study	Primary dentition; HSPM-affected and unaffected second primary molars	HSPM	Enamel	10 HSPM; 10 controls	4–8	mDDE/EAPD index; investigators trained in HSPM diagnosis (κ = 0.6)	Opacities and post-eruptive breakdown; deep caries permitted; atypical restorations or extensive enamel destruction excluded
Singh et al. [20]	India	In vitro comparative study	Primary dentition; HSPM-affected and unaffected second primary molars	HSPM	Enamel	10 HSPM; 10 controls	NR	EAPD criteria	White, yellow, brown
Singh et al. [22]	India	Secondary comparative proteomic analysis	Permanent dentition; first permanent molars affected by MIH and sound enamel, Primary dentition; HSPM-affected and unaffected second primary molars	HSPM and MIH	Enamel	10 HSPM; 10 MIH; final analysis: 12 (6/group)	NR	EAPD criteria	NR
Salivary proteomics									
Bekes et al. [12]	Austria	Observational analytical cross-sectional study	Permanent dentition; saliva from children with MIH and healthy controls	MIH	Saliva	5 MIH; 5 controls	6–14	EAPD criteria	MIH-TNI
Pappa et al. [14]	Greece	Observational analytical cross-sectional study	Permanent dentition; saliva from children with MIH and healthy controls	MIH	Saliva	20 participants (10 MIH; 10 healthy controls)	Mean age: MIH 14.8 ± 1.7 years; Controls 15.2 ± 1.8 years	EAPD criteria	NR
Toledo et al. [21]	Brazil	Cross-sectional study	Permanent dentition; saliva from children with MIH and healthy controls.	MIH	Saliva	50 children, allocated into five groups (n = 10/group): Control, Mild MIH (white/cream opacity), Mild MIH (yellow/brown opacity), Severe MIH without caries, and Severe MIH with caries.	8–13	EAPD criteria	Mild/severe

Abbreviations: MIH, molar–incisor hypomineralisation; HSPM, hypomineralised second primary molars; MH, molar hypomineralisation; EAPD, European Academy of Paediatric Dentistry; MIH-TNI, Molar–Incisor Hypomineralisation Treatment Need Index; mDDE, modified Developmental Defects of Enamel index; ALB, albumin; AFP, alpha-fetoprotein; NR, not reported.

**Table 2:** Proteomic characteristics and main findings of the included studies.

Author (Year)	Analytical Methodology	Proteomic Findings	Differentially Abundant Proteins	Biological Pathways	Principal Findings	Funding
Enamel proteomics						
Farah et al. [10]	Protein extraction (20% TCA), SDS-PAGE, LC–MS/MS (LTQ-Orbitrap XL), protein identification using Mascot (UniProt/SWISS-PROT)	Serum-derived proteins and enamel-related proteins were detected across sound and MIH enamel, with qualitative differences according to lesion severity	Higher abundance in yellow and brown MIH enamel: serum albumin (ALB) and α1-antitrypsin (SERPINA1). Detected only in yellow and brown MIH enamel: antithrombin III (SERPINC1). Detected only in brown MIH enamel: ameloblastin (AMBN; at the limit of detection)	NA (targeted detection study)	MIH enamel contained 8–21-fold more protein than sound enamel, particularly in brown lesions. Increased serum-derived proteins (especially albumin and α1-antitrypsin) suggest that serum infiltration contributes to impaired enamel maturation	Partly funded by a grant from the New Zealand Dental Association Research Foundation
Williams et al. [16]	Western blot (ALB and AFP), hydroxyapatite-binding assays and KLK4 digestion assays.	Adult serum albumin (ALB) and α-fetoprotein (AFP) were detected in hypomineralised enamel. Both albumin isoforms were identified in six of eight chalky opacities, corresponding to four of five cases	Not applicable—no differential proteomic analysis was performed	NA (targeted detection study)	Foetal albumin was detected in intact hypomineralised enamel, supporting developmental incorporation of serum-derived proteins. Albumin/AFP showed hydroxyapatite binding and resistance to KLK4 digestion.	Melbourne Research Unit for Facial Disorders (MH, JM, RW and VP), Department of Pharmacology & Therapeutics (MH, JM, RW, and VP), Department of Paediatrics, and Faculty of Medicine, Dentistry and Health Science (MH) at the University of Melbourne. JM held a Peter Doherty early career fellowship from NHMRC, Australia. VP additionally received Ph.D. scholarship support in Melbourne from Becas Chile and the University of Talca where he now holds a faculty position.
Mukhtar et al. [13]	Acid-etched enamel proteins analysed by label-free LC–MS/MS (Orbitrap Fusion Tribrid). Protein identification using Proteome Discoverer (UniProt), followed by GO, KEGG, DAVID, IMPaLA and STRING analyses	271 proteins identified overall: 76 exclusive to MIH, 39 exclusive to sound enamel, and 156 common to both groups. MIH enamel contained significantly more proteins than sound enamel (50.3 ± 29.6 vs 21.4 ± 3.2 proteins/sample).	Higher abundance in MIH enamel: albumin (ALB), dentin sialophosphoprotein (DSPP), antithrombin III (SERPINC1), calcium-binding proteins, immunoglobulins, S100A8/S100A9 and inflammatory/stress-response proteins. Higher abundance in sound enamel: collagens, α1-antitrypsin (SERPINA1), KLK4, MMP20, α2-macroglobulin (A2M) and α2-HS-glycoprotein (AHSG)	Innate immune response, inflammatory response, neutrophil aggregation, chemokine production, fibrinolysis, collagen biosynthesis, blood coagulation and Toll-like receptor signalling	MIH enamel exhibited a distinct proteomic profile characterised by increased serum-derived and inflammatory proteins and reduced enamel maturation proteins. These findings suggest impaired mineralisation associated with protease/anti-protease imbalance and persistent albumin interfering with hydroxyapatite crystal growth.	No
Rexhaj et al. [15]	FASP protein extraction, TMT-based LC–MS/MS (Orbitrap Fusion Tribrid), protein identification using Proteome Discoverer/Mascot (SwissProt), followed by differential expression analysis	Compared with sound enamel, 179 proteins were differentially abundant in MIH I and 60 in MIH II	Higher abundance in MIH enamel: annexin A2 (ANXA2), plakophilin-1 (PKP1), desmoplakin (DSP), junction plakoglobin (JUP), several keratins and small proline-rich proteins. Lower abundance in MIH enamel: proteins mainly associated with cell organisation, biogenesis, metabolism and defence response. Profiles differed between white/creamy and yellow/brown lesions	Cell death, cell organisation and biogenesis, metabolic processes and defence response	Proteomic analysis differentiated healthy enamel from MIH enamel and distinguished MIH severity grades. MIH enamel showed increased proteins related to cell death and reduced proteins involved in cell organisation and biogenesis, with distinct profiles between white/creamy and yellow/brown lesions.	Open access funding provided by University of Gothenburg. This study was supported by grants from TUA Research Funding at the Sahlgrenska Academy, University of Gothenburg, Region Västra Götaland, Sweden.
Sharon et al. [19]	Bradford assay; SDS-PAGE and 2D-gel electrophoresis; MALDI-TOF/TOF MS	Twenty-one proteins were significantly upregulated in HSPM enamel compared with normal enamel, involving serum-derived, enamel matrix, protease-inhibitory, immune and cellular proteins	Upregulated in HSPM enamel included DSPP, serum albumin (ALB), antithrombin III (SERPINC1), α1-antitrypsin (SERPINA1), vitamin D-binding protein, apolipoprotein A-I and immune-related proteins (*p* < 0.05)	Enamel matrix organisation, biomineralisation, proteolysis, immune response and protein–protein interaction networks	HSPM enamel contained markedly more protein than normal enamel, supporting reduced proteolysis and increased protease-inhibitor activity as possible mechanisms	No specific funding
Singh et al. [20]	Label-free LC–MS/MS (Orbitrap Fusion), protein identification using Proteome Discoverer/Sequest HT, pathway analyses and Western blot/dot-blot validation	The mean number of proteins identified was significantly higher in HSPM enamel than in control enamel (134.9 ± 14.8 vs 71.7 ± 14.2 proteins per sample). 77 proteins were upregulated in HSPM enamel, whereas 73 proteins were more abundant in control enamel	Higher abundance in HSPM enamel included SERPINB1, KLK1, HTRA1, albumin, calcium-binding proteins and immunoglobulins; control enamel showed higher abundance of proteins related to matrix organisation and mineralisation, including LAMA3, FGFR1, osteopontin, matrix Gla protein and several collagens.	Complement and coagulation cascades, neutrophil and platelet degranulation, innate immune response, antimicrobial defence and inflammatory response	HSPM enamel contained significantly more proteins and showed enrichment of immune/inflammatory pathways, whereas control enamel retained more proteins involved in normal matrix organisation and mineralisation	PGIMER,IM/305/21-03-23-0832,Manojkumar Jaiswal
Singh et al. [22]	Label-free quantitative LC–MS/MS (Orbitrap Fusion Tribrid); Proteome Discoverer v2.4; UniProt; STRING/GO/KEGG/Reactome pathway analysis	435 proteins identified; 206 unique to HSPM, 109 unique to MIH, and 120 shared; 90 shared proteins retained for quantitative analysis	46 proteins (FDR < 0.05): 38 enriched in HSPM and 8 enriched in MIH	HSPM: acute-phase/inflammatory response, proteolysis regulation, complement/coagulation, platelet/neutrophil degranulation; MIH: keratinocyte differentiation, keratinisation/cornified envelope, cytoskeletal organisation, apoptosis and oxidative-stress/immune pathways	HSPM and MIH showed a largely shared enamel proteome but distinct quantitative profiles; HSPM was predominantly enriched in immune/plasma and protease–antiprotease-related proteins, whereas MIH showed greater enrichment of epithelial/cornification-related proteins	NR
Salivary proteomics						
Bekes et al. [12]	Protein extraction and tryptic digestion, label-free LC–MS/MS (Q-Exactive Plus), MaxQuant, GO/KEGG/Reactome enrichment and STRING network analysis	618 proteins identified; 88 proteins exclusive to MIH and 16 exclusive to controls.	Proteins exclusive to MIH included CFI, CD55, IL18, IL36RN, KLK8, PGLYRP2, ORM2, S100A8 and S100A9. Exclusive to controls: 16 proteins	Complement activation, innate immune response, inflammatory response, neutrophil degranulation, platelet degranulation and lysosomal pathways	MIH saliva exhibited a distinct inflammatory proteomic signature, with activation of innate immune pathways, complement cascade and neutrophil-related proteins, suggesting a chronic inflammatory oral environment	NR
Pappa et al. [14]	FASP digestion, nanoLC–MS/MS (Q Exactive HF-X Orbitrap), Proteome Discoverer/SequestHT and MetaLab/MaxQuant, followed by GO enrichment and microbial taxonomic profiling	1515 high-confidence human proteins identified; 142 proteins were differentially expressed between MIH and controls. Metaproteomic analysis revealed reduced microbial diversity in the MIH group	Higher abundance in MIH saliva: immunoglobulin-related proteins, complement C5, plasminogen, annexin A1, S100A8/S100A9 and other inflammatory and innate immune proteins. Lower abundance in MIH saliva: proteins involved in antimicrobial defence, antioxidant activity and salivary homeostasis.	Complement activation, acute inflammatory response, innate immune response, antioxidant defence and microbial dysbiosis	Salivary proteomics clearly discriminated MIH patients from healthy controls. Dysregulation of inflammatory and immune pathways and reduced microbial diversity support saliva as a potential source of diagnostic biomarkers.	No
Toledo et al. [21]	Label-free LC–MS/MS following tryptic digestion, with GO, KEGG, Reactome and STRING bioinformatic analyses	6471 proteins were identified. 5073 proteins were exclusive to MIH groups and 778 proteins were shared among different MIH severity groups. Distinct proteomic signatures were observed according to disease severity.	Severity-dependent differences were observed in the salivary proteome. Severe MIH showed higher abundance of proteins related to calcium-ion binding, microtubule binding and platelet-derived growth factor binding; individual differentially abundant proteins were not consistently reported across all pairwise group comparisons	Calcium-ion binding, platelet-derived growth factor binding, microtubule binding, cytoskeletal organisation, cell adhesion and immune-related pathways	Salivary proteomic profiles differed according to MIH severity, demonstrating progressive molecular changes from mild to severe lesions. The findings suggest that saliva contains distinct proteomic signatures according to MIH severity that may contribute to biomarker discovery and improve understanding of MIH pathophysiology and clinical progression.	No

Abbreviations: MIH, molar–incisor hypomineralisation; HSPM, hypomineralised second primary molars; MH, molar hypomineralisation; NR, not reported; NA, not applicable; LC–MS/MS, liquid chromatography–tandem mass spectrometry; SDS-PAGE, sodium dodecyl sulphate–polyacrylamide gel electrophoresis; MALDI-TOF/TOF MS, matrix-assisted laser desorption/ionisation tandem time-of-flight mass spectrometry; FASP, filter-aided sample preparation; TMT, tandem mass tags; TCA, trichloroacetic acid; FDR, false discovery rate; GO, Gene Ontology; KEGG, Kyoto Encyclopedia of Genes and Genomes; ALB, albumin; AFP, alpha-fetoprotein.

## Data Availability

No new data were created or analysed in this study. All data supporting the findings of this systematic review are available in the included studies and the Supplementary Materials.

## Supplementary Materials

The following supporting information can be downloaded at: https://www.mdpi.com/article/10.3390/dj14100681/s1, Table S1: Search strategies used across the electronic databases; Table S2: Studies excluded after full-text assessment and reasons for exclusion; Table S3: Methodological appraisal of the included studies using the JBI Critical Appraisal Checklist and the Quality Assessment Tool for In Vitro Studies (QUIN); Table S4: Integrated synthesis of proteomic findings and their potential biological relevance in developmental enamel hypomineralisation; Table S5: Completed PRISMA 2020 reporting checklist.

## Abbreviations

The following abbreviations are used in this manuscript:

MH	Molar Hypomineralization
MIH	Molar Incisor Hypomineralization
HSPM	Hypomineralization Second Primary Molars
EAPD	European Academy Pediatric Dentistry
