# Supplementary material for: Proteomic Alterations in Hypomineralised Enamel: A Systematic Review
Source: Dent J (Basel). 2026 Oct 19;14(10):681. doi: 10.3390/dj14100681 (PMC13703422; doi:10.3390/dj14100681)
Supplement: Supplementary file 1 [file dentistry-14-00681-s001.zip › dentistry-4597626-supplementary.pdf]

**Supplementary Table S1. Search strategies used across the electronic databases**

| | |
|---|---|
| PubMed (MEDLINE) | ("Molar Incisor Hypomineralization"[Mesh] OR "molar incisor hypomineralization" OR "molar-incisor hypomineralisation" OR MIH) AND (Proteomics[Mesh] OR proteom* OR protein* OR biomarker* OR peptide* OR "mass spectrometry" OR<br>"liquid chromatography-mass spectrometry" OR LC-MS OR LC-MS/MS OR MALDI) |
| Embase | ("molar incisor hypomineralization" OR "molar hypomineralization" OR "molar-incisor hypomineralisation" OR "MIH") AND ("proteomics" OR "proteom*" OR "protein*" OR "biomarker*" OR "peptide*" OR "mass spectrometry" OR "liquid chromatography" OR "LC-MS" OR "LC-MS/MS" OR "MALDI") |
| Scopus | TITLE-ABS-KEY<br>("molar incisor hypomineralization" OR "molar-incisor hypomineralisation" OR MIH) AND (proteom* OR protein* OR biomarker* OR peptide* OR "mass spectrometry" OR LC-MS OR LC-MS/MS OR MALDI) |
| Web of Science | TS=<br>("molar incisor hypomineralization" OR "molar-incisor hypomineralisation" OR MIH) AND (proteom* OR protein* OR biomarker* OR peptide* OR "mass spectrometry" OR LC-MS OR LC-MS/MS OR MALDI) |

**Supplementary Table S2. Studies excluded after full-text assessment and reasons for exclusion**

| **Reference** | **Exclusion criterion** | **Specific reason** |
|---|---|---|
| Lavôr JM, Fernandes IC, Brito DHS, Sampaio FC, Rosenblatt A, Vieira AR. *Insights into AQP5 Polymorphism and Genetic Associations in Molar Incisor Hypomineralization and Dental Fluorosis*. Caries Research. 2026;60(3):249–261. | Wrong outcome | Genetic association study |
| Timoshenko PS, Korolenkova MV. *Efficiency of permanent teeth filling in children with molar-incisor hypomineralization*. Stomatology. 2025;104(5):79–83. | Wrong outcome | Clinical treatment study |
| Sahin S, Oktay NS, Caliskan S, Kargul B, Sen Yavuz B. *Assessment of Inflammatory Cytokines in Molar Incisor Hypomineralization-affected First Permanent Molars: A Clinical Study*. Journal of Endodontics. 2026. | Wrong outcome | Cytokine analysis |
| Tok A, Altınsoy N, Hoşnut FÖ. *Investigation of the frequency of molar incisor hypomineralisation in childhood celiac disease and evaluation with nutritional factors and calcium metabolism*. Frontiers in Pediatrics. 2025;13:1603751. | Wrong outcome | Epidemiological association study |
| Wang L, Mosquim V, Zabeu GS, Feltrin-Souza J. *MIH-Affected Substrate: An Overview of Adhesive Challenges to the Dental Structures*. Monographs in Oral Science. 2024;32:195–211. | Review article | Narrative review |
| Jorge RC, Reis PPGD, Soviero VM. *Understanding the Enamel Disintegration and Posteruptive Breakdown of Demarcated Opacities on Molar Incisor Hypomineralisation*. Monographs in Oral Science. 2024;32:88–99. | Review article | Narrative review |
| Gonçalves JL, Carvalho FK, Queiroz AM, Paula-Silva FWG. *Implications of Histological and Ultrastructural Characteristics on the Chemical and Mechanical Properties of Hypomineralised Enamel and Clinical Consequences*. Monographs in Oral Science. 2024;32:43–55. | Review article | Narrative review |

| Reference | Exclusion criterion | Specific reason |
|---|---|---|
| Luppieri V, Bayrak GD, Buttazzoni T, Tabakcilar D, et al. *Prevalence and Clinical Patterns of Molar-Incisor Hypomineralisation (MIH) and Hypomineralised Second Primary Molars (HSPM) in Italian and Turkish Children Aged 6–16 Years: An Epidemiological Comparative Cross-Sectional Study.* European Journal of Paediatric Dentistry. 2025. | Wrong outcome | Epidemiological study |
| Den Besten PK, Diekwisch TGH. *Editorial: Tooth Enamel Research: Enamel 10 and Beyond*. Frontiers in Physiology. 2023;14:1323504. | Editorial | Editorial |
| Elzein R, Abdel-Sater F, Mehawej C, Jalkh N, Ayoub F, Chouery E. *Identification by Whole-Exome Sequencing of New Single-Nucleotide Polymorphisms Associated with Molar-Incisor Hypomineralisation among the Lebanese Population*. European Archives of Paediatric Dentistry. 2022;23:919–928. | Wrong outcome | Genetic association study |
| Yang J, Huang T, Yao J, Zhang J, Bai G, Chen Z, Tu C. *Sulphur Dioxide and Fluoride Co-exposure Induce Incisor Hypomineralization and Amelogenin Upregulation via YAP/RUNX2 Signaling Pathway*. Ecotoxicology and Environmental Safety. 2022;245:114106. | Wrong outcome | Experimental signalling study |
| Babajko S, Jedeon K, Houari S, Loiodice S, Berdal A. *Disruption of Steroid Axis, a New Paradigm for Molar Incisor Hypomineralization*. Frontiers in Physiology. 2017;8:343. | Wrong outcome | Aetiological hypothesis |
| Børsting T, Schuller A, van Dommelen P, et al. *Maternal Vitamin D Status in Pregnancy and Molar Incisor Hypomineralisation and Hypomineralised Second Primary Molars in the Offspring at 7–9 Years of Age: A Longitudinal Study*. European Archives of Paediatric Dentistry. 2022;23:557–566. | Wrong outcome | Vitamin D association study |
| Vieira AR, Kup E. *On the Etiology of Molar-Incisor Hypomineralization*. Caries Research. 2016;50:166–169. | Review article | Narrative review |

| Reference | Exclusion criterion | Specific reason |
|---|---|---|
| Farah RA, Drummond BK, Swain MV, Williams S. *Relationship between Laser Fluorescence and Enamel Hypomineralisation*. Journal of Dentistry. 2008;36:915–921. | Wrong outcome | Laser fluorescence study |
| Jedeon K, De la Dure-Molla M, Brookes SJ, et al. *Enamel Defects Reflect Perinatal Exposure to Bisphenol A*. American Journal of Pathology. 2013;183:108–118. | Animal study | Rat model |
| Rodd HD, Morgan CR, Day PF, Boissonade FM. *Pulpal Expression of TRPV1 in Molar Incisor Hypomineralisation*. European Archives of Paediatric Dentistry. 2007;8:184–188. | Wrong outcome | Pulpal biomarker study |
| van der Tas JT, Elfrink MEC, Heijboer AC, et al. *Foetal, Neonatal and Child Vitamin D Status and Enamel Hypomineralization*. Community Dentistry and Oral Epidemiology. 2018;46:343–351. | Wrong outcome | Vitamin D association study |
| Memarpour M, Shafiei F, Rafiee A, Soltani M, Dashti MH. *Effect of Hydroxyapatite Nanoparticles on Enamel Remineralization and Estimation of Fissure Sealant Bond Strength to Remineralized Tooth Surfaces*. BMC Oral Health. 2019;19:92. | Wrong outcome | Remineralization study |
| Dhamo B, Miliku K, Voortman T, et al. *The Associations of Maternal and Neonatal Vitamin D with Dental Development in Childhood*. Current Developments in Nutrition. 2019;3:nzy100. | Wrong outcome | Tooth development study |
| Çetin PR, Alkan E, Tağtekin D, Çetin Aİ, Oktay NŞ. *Enamel Remineralization Potential of Bioactive Glass-containing Toothpastes: An In Vitro Study*. Odontology. 2026. | Wrong outcome | Remineralization study |
| Bilgir BS, Aydınoğlu S, Arslan İ, et al. *Analysis of MMP-8, MMP-20, and TGF-β1 in Molar-Incisor Hypomineralization and Assessment of Periodontal Health*. BMC Oral Health. 2025;25:1189. | Wrong outcome | Periodontal biomarkers |
| Gomes-Souza L, Fatturi AL, Scariot R, et al. *Association between Multiple Genetic Polymorphisms and Molar-Incisor Hypomineralization: A Population-based Study*. Journal of Applied Oral Science. 2025;33:e20250074. | Wrong outcome | Genetic association study |

| **Reference** | **Exclusion criterion** | **Specific reason** |
|---|---|---|
| Chun YP, Tan C, Villanueva O, et al. *Overexpression of Ameloblastin in Secretory Ameloblasts Results in Demarcated, Hypomineralized Opacities in Enamel*. Frontiers in Physiology. 2024;14:1233391. | Animal study | Mouse model |
| Pang L, Li X, Wang K, et al. *Interactions with the Aquaporin 5 Gene Increase the Susceptibility to Molar-Incisor Hypomineralization*. Archives of Oral Biology. 2020;111:104637. | Wrong outcome | Genetic association study |
| Jedeon K, Marciano C, Loiodice S, et al. *Enamel Hypomineralization Due to Endocrine Disruptors*. Connective Tissue Research. 2014;55(Suppl 1):43–47. | Animal study | Animal model |
| Porto IM, Laure HJ, Sousa FB, Rosa JC, Gerlach RF. *New Techniques for the Recovery of Small Amounts of Mature Enamel Proteins*. Journal of Archaeological Science. 2011;38:3596–3604. | Wrong outcome | Methodological study |
| Mortensen NB, Haubek D, Dalgård C, et al. *Vitamin D Status and Tooth Enamel Hypomineralization Are Not Associated in 4-Year-Old Children: An Odense Child Cohort Study*. Journal of Steroid Biochemistry and Molecular Biology. 2022;221:106130. | Wrong outcome | Vitamin D association study |
| S M, N M, C JM, et al. *DNA Methylation in Childhood Dental Caries and Hypomineralization*. Journal of Dentistry. 2022;117:103913. | Wrong outcome | Epigenetic study |
| Mangum JE, Veith PD, Reynolds EC, Hubbard MJ. *Towards Second-generation Proteome Analysis of Murine Enamel-forming Cells*. European Journal of Oral Sciences. 2006;114(Suppl 1):259–265. | Animal study | Murine model |
| Malmberg P, Norén JG, Bernin D. *Molecular Insights into Hypomineralized Enamel*. European Journal of Oral Sciences. 2019;127:340–346. | Wrong outcome | Molecular composition analysis (not proteomics) |

| **Reference** | **Exclusion criterion** | **Specific reason** |
|---|---|---|
| Castiblanco GA, Rutishauser D, Ilag LL, Martignon S, Castellanos JE, Mejía W. *Identification of Proteins from Human Permanent Erupted Enamel*. European Journal of Oral Sciences. 2015;123:390–395. | Wrong population | Healthy enamel only |
| Fatturi AL, Menoncin BL, Reyes MT, et al. *The Relationship between Molar Incisor Hypomineralization, Dental Caries, Socioeconomic Factors, and Polymorphisms in the Vitamin D Receptor Gene: A Population-based Study*. Clinical Oral Investigations. 2020;24:3971–3980. | Wrong outcome | Genetic association study |
| Gil-Bona A, Karaaslan H, Depalle B, et al. *Proteomic Analyses Discern the Developmental Inclusion of Albumin in Pig Enamel: A New Model for Human Enamel Hypomineralization*. International Journal of Molecular Sciences. 2023;24:15577. | Animal study | Pig model |
| Horta HF, Sampaio LV, Barzotti RJ, et al. *Salivary Changes Associated with Hypomineralized Second Primary Molars (HSPM) and Molar Incisor Hypomineralization (MIH): An Early Study*. Journal of Dentistry. 2026;164:106230. | Wrong outcome | Salivary biochemical biomarkers (not proteomics) |
| Jeremias F, Koruyucu M, Küchler EC, et al. *Genes Expressed in Dental Enamel Development Are Associated with Molar-Incisor Hypomineralization*. Archives of Oral Biology. 2013;58:1434–1442. | Wrong outcome | Genetic association study |
| Rexhaj F, Sabel N, Robertson A, Lundgren T. *Evaluation of Method Parameters for Sound Undecalcified Dental Enamel Proteomics Using Liquid Chromatography-Mass Spectrometry*. Archives of Oral Biology. 2023;155:105802. | Wrong population | Healthy enamel only |
| Tynior W, Hudy D, Gołąbek K, et al. *Expression of AMELX, AMBN, ENAM, TUFT1, FAM83H and MMP20 Genes in Buccal Epithelial Cells from Patients with Molar Incisor Hypomineralization (MIH): A Pilot Study*. International Journal of Molecular Sciences. 2025;26:766. | Wrong outcome | Gene expression study |

| Reference | Exclusion criterion | Specific reason |
|---|---|---|
| Yang N, Fan J, Man G, Wang W, Liao J, Tu C. *Proteomic Signatures of Dental Enamel Hypomineralization: Divergent Molecular Pathways in Coal-Burning vs. Drinking-Water Fluorosis*. Biological Trace Element Research. 2026;204:3152–3167. | Wrong population | Dental fluorosis |
| Zhou M, Jia X, Wan H, Wang S, Zhang X, Zhang Z, Wang Y. *miR-34 Regulates Reproduction by Inhibiting the Expression of MIH, CHH, EcR, and FAMeT Genes in Mud Crab Scylla paramamosain*. Molecular Reproduction and Development. 2019;86:122–131. | Wrong topic | MIH = molt-inhibiting hormone (not molar-incisor hypomineralization) |

**Supplementary Table S3. Methodological appraisal of the included studies using the JBI Critical Appraisal Checklist and the Quality Assessment Tool for In Vitro Studies (QUIN)**

**Table S3A. Risk-of-bias assessment of analytical cross-sectional studies using the revised JBI tool**

| Study | Q1 | Q2 | Q3 | Q4 | Q5 | Q6 | Q7 | Q8 | Overall judgement |
|---|---|---|---|---|---|---|---|---|---|
| Bekes et al. (2020) | Yes | Yes | Yes | Yes | No | No | Yes | Yes | Some concerns |
| Pappa et al. (2022) | Yes | Yes | Yes | Yes | Yes | Yes | Yes | Yes | Low risk |
| Toledo et al. (2025) | Yes | Yes | Yes | Yes | Yes | Yes | Yes | Unclear | Some concerns |

**JBI items:** Q1, criteria for inclusion in the sample clearly defined; Q2, study subjects and setting described in detail; Q3, exposure measured in a valid and reliable way; Q4, objective, standard criteria used for measurement of the condition; Q5, confounding factors identified; Q6, strategies to deal with confounding factors stated; Q7, outcomes measured in a valid and reliable way; and Q8, appropriate statistical analysis used.

**Table S3B. Risk-of-bias assessment of laboratory-based studies using the Quality Assessment Tool for In Vitro Studies (QUIN)**

| Study | Q1 | Q2 | Q3 | Q4 | Q5 | Q6 | Q7 | Q8 | Q9 | Q10 | Q11 | Q12 | Overall score (%) | Risk of bias |
|---|---|---|---|---|---|---|---|---|---|---|---|---|---|---|
| **Farah et al., 2010** | 2 | 0 | 1 | 2 | 2 | 0 | 1 | 2 | 0 | 0 | 1 | 2 | 54.2 | Medium risk |
| **Mukhtar et al., 2022** | 2 | 1 | 1 | 2 | 2 | 1 | 0 | 2 | 0 | 0 | 2 | 2 | 62.5 | Medium risk |
| **Rexhaj et al., 2024** | 2 | 0 | 1 | 2 | 2 | 1 | 0 | 2 | 0 | 0 | 2 | 2 | 58.3 | Medium risk |
| **Williams et al., 2020** | 2 | 0 | 1 | 2 | 2 | 1 | 0 | 2 | 0 | 0 | 1 | 2 | 54.2 | Medium risk |
| **Sharon et al., 2025** | 2 | 1 | 2 | 2 | 2 | 1 | 0 | 2 | 0 | 0 | 2 | 2 | 66.7 | Medium risk |
| **Singh et al., 2025** | 2 | 1 | 2 | 2 | 2 | 1 | 0 | 2 | 0 | 0 | 2 | 2 | 66.7 | Medium risk |
| **Singh et al., 2026** | 2 | 0 | 2 | 2 | 2 | 1 | 0 | 2 | 0 | 0 | 2 | 2 | 62.5 | Medium risk |

**QUIN criteria:** Q1, Aim/objective stated; Q2, Sample-size calculation; Q3, Sampling technique described; Q4, Comparator group described; Q5, Methodology explained in detail; Q6, Operator details; Q7, Randomization; Q8, Measurement method described; Q9, Outcome assessor details; Q10, Blinding; Q11, Statistical analysis; Q12, Results presentation.

**Scoring:** 2 = adequately specified; 1 = inadequately specified; 0 = not specified; N/A = not applicable and excluded from the denominator. Overall score = (raw score / maximum applicable score) × 100. Risk of bias: >70% = low; 50–70% = medium; <50% = high.

**Supplementary Table S4**. Integrated synthesis of proteomic findings and their potential biological relevance in developmental enamel hypomineralisation.

| Proteomic domain | Main findings across studies | Potential biological relevance |
|---|---|---|
| Serum-derived proteins | Recurrent detection/retention of albumin and other serum-derived proteins in hypomineralised enamel; albumin was among the most consistently reported findings. | Abnormal retention of serum proteins during enamel maturation; compatible with interference with crystal growth and mineralisation. |
| Protease–antiprotease balance | Alterations in protease inhibitors and enamel proteases, including α1-antitrypsin, MMP20 and KLK4; HSPM also showed enrichment of several serine protease inhibitors. | Potential imbalance between proteolytic activity and protein removal during enamel maturation. |
| Enamel matrix and maturation proteins | Alterations in proteins associated with enamel matrix processing and maturation, including MMP20, KLK4, ameloblastin and related proteins. | Consistent with altered matrix processing and impaired enamel maturation. |
| Immune and inflammatory response | Differential abundance of proteins associated with innate and adaptive immunity and inflammatory responses in enamel and saliva; immune-related proteins were particularly enriched in HSPM in the direct HSPM–MIH comparison. | Suggests involvement of immune/inflammatory processes, although their developmental versus post-eruptive origin remains uncertain. |
| Complement/coagulation pathways | Alterations in complement- and coagulation-related proteins, with enrichment of these pathways particularly evident in HSPM. | May reflect inflammatory/systemic protein incorporation or post-eruptive infiltration into porous hypomineralised enamel. |

| Proteomic domain | Main findings across studies | Potential biological relevance |
|---|---|---|
| Mineralisation/calcium-related processes | Alterations in calcium-binding and mineralisation-associated proteins. | Potential disturbance of mineral homeostasis and enamel mineralisation. |
| Epithelial/cornification and cellular processes | Alterations in extracellular matrix and cellular proteins; MIH showed enrichment of keratins, cornification-associated and desmosomal proteins in the direct HSPM–MIH comparison. | May reflect altered epithelial differentiation, cytoskeletal organisation and enamel-organ processes; post-eruptive incorporation cannot be excluded. |
| Severity-associated proteomic changes | Proteomic profiles differed according to clinical severity in some enamel and salivary studies. | Suggests that molecular alterations may vary with phenotypic severity. |
| Patterns across dentitions | HSPM and MIH showed a largely shared protein repertoire but distinct quantitative proteomic profiles; HSPM was relatively enriched in immune/plasma and protease–antiprotease-related proteins, whereas MIH showed greater enrichment of epithelial/cornification-related proteins. | Suggests overlapping molecular features across dentitions alongside potentially condition- or dentition-specific quantitative differences. |
